# Epigenetics, society, and bio-objects

**DOI:** 10.3325/cmj.2015.56.166

**Published:** 2015-04

**Authors:** Anna Lydia Svalastog, Maria Damjanovicova

**Affiliations:** 1 Østfold University College, Norway; 2 Centre for Research Ethics and Bioethics, Uppsala University, Uppsala, Sweden; 3 European Institute of Oncology, Milan, Italy maria.damjanovicova@ieo.eu; * Both authors equally contributed to this text.

The molecular account of the environmental that epigenetics offers bestow it with paramount importance for biomedical perspective of health and disease and for social sciences perspective on human interactions and well-being. It offers new prospects for interventions to shape the health of both individuals and populations, and invokes its own ethical, legal, and social implications. We here propose treatment of the relation between epigenetics and society through the framework of bio-objectification. After discussing how epigenetic information becomes part of particular bio-identification and bio-objectification processes, we then propose the bio-objectification framework as a fruitful conceptual framework for the analysis of dynamic research and responsive regulatory landscape of this biotechnology.

“What counts more: nature or nurture, the biological or the social” has long been occupying minds of various people, from philosophers, to social and natural scientists, to politicians and physicians, as well as of common citizens. Although much has been said on this topic from an “interactionist” point of view (both aspects, biological/nature and social/nurture, are important), the “divide” survived into contemporary days with “wars” being fought on whether it is the DNA or the environment that make us who we are. In this sense, the cover of the Time Magazine “Why your DNA isn’t your destiny” (1) epitomizes “the new interactionist” paradigm brought about by rapid developments in the field of epigenetics. With reference to what is on or above the genome (*epi*- in Greek), “epigenetics collectively describes changes in the regulation of gene expression that can be passed on to a cell’s progeny but are not due to changes to the nucleotide sequence of the gene” (2). These changes, depicted through epigenetic marks, “determine which genes are expressed by which cell type, and when” (2) and are transducers of the environments in which genes prevail. And as technological developments make possible the study of more and more complex interactions of genes and their environments, the “gene context” (in genetic science, considered as the cellular context of the genes) gets extended beyond the cellular level to tie variations in gene expression between individuals with social variables like socio-economic status (3,4) and industrialization- and urbanization-produced life styles (5). Such molecular account of the social grants epigenetics paramount importance. It influences, on the one hand, biomedical perspective of health and disease, and, on the other hand, social sciences perspective on human interactions and well-being. Molecular visibility (6) of such wide spectrum of effects, from environmental pollution to parental care, to individual lifestyles or socio-economic status, proposed by epigenetics, together with the plasticity of epigenetic marks, offers new prospects for interventions to shape the health of both individuals and populations.

One proposal as to how epigenetics promises to deliver these prospects relies on causality: epigenetics will finally allow to causally link environmental and social exposures to bodily and health outcomes. Once the cascade of causal events that leads to poor health outcomes becomes dissected, it will be possible to interfere with these processes to: 1) provide better treatment as well as diagnostic and prognostic tools, and 2) develop effective preventive strategies. This “causal” reading of epigenetics bears great implications for responsibility for health. In cases of undesirable health outcomes and allocation of limited health care resources, it could tip the balance between the individual responsibility for “poor health-related behaviour” and society and community responsibility for providing enough opportunities for “good health-related behaviour” (7). Tipping the balance between individual and collective responsibility inevitably invites questions of what counts as good and poor health-related behavior and how to disentangle multitude of factors. For example, when the same individual that does not smoke but practices very bad eating habits (which can indeed reflect either their personal choice to eat unhealthy, but might be influenced by the lower economic status or work related conditions), which of all these factors should be considered in deciding the factor that caused undesirable health outcome? The question of whose responsibility the lived life represents is then closely tied to questions of how to establish risk categories and how to stratify people across these categories according to their health-related behavior (8). And if the social is of major importance to gene expression, how do we weight social patterns of behavior or dietary patterns that strengthen well-being and community?

The epigenetics promises to deliver the above mentioned prospects through digitization: epigenetics is a *transformer of analogical vastness* of the environmental and the social into *digital, code- and* therefore *genome-compatible information* (9). On this perspective, a new layer of complexity is added to the existing debates on bio-data management, with one of the leading concerns being related to identifiability of patients and research participants in cases of privacy breaches into databases (10,11). As potentially sensitive information about individuals’ private lifestyles becomes digitized in epigenomic data sets, it is digitally linked to their unique and therefore identifiable genomic data, some of which are stored in publically accessible databases. After listing just few of the most prominent ethical, legal and social concerns (12,13), we here attempt treatment of the relation between epigenetics and society through the framework of bio-objectification. In discussing how biological material (biomaterial) and information obtained from tinkering with that material (bio-information) become part of particular *bio-identification* (analytical process of asking what new relations novel bio-objects generates and/or upset, and how they are/could be governed) and potentially *bio*-*objectification* processes (analysis of the ways in which novel bio-objects are created and ‘natural’ boundaries they disturb), we propose the bio-objectification framework as a fruitful conceptual framework for the analysis of dynamic research and responsive regulatory landscape of biotechnology.

The concepts of bio-objects, bio-objectification and bio-identification have been launched and elaborated within the frame of the COST Action IS 1001 as new tools for analysis of present bio-technology products (novel biological materials and forms of life), their production processes, and their social, economic and cultural implications and consequences (14). Bio-objects as entities *move between* institutional, regulatory and legal boarders of research, medicine, food, agriculture and industry (15,16). There are epigenetic research outputs whose potential bio-objectification value has been anticipated by their technological predecessor: 1) epigenetically reprogrammed cell types (induced pluripotent stem cells, iPSCs, and their differentiated derivatives), which can be used for both replacement therapy and drug discovery process (17), and 2) predictive epigenetic profiles, which will require (due to above mentioned complex nature of epigenetic marks – namely plasticity in relation to environmentally and socially induced changes) prospective epigenome sampling and deposition of source tissues and cells in biorepositories. Since both of these outputs, iPSCs and predictive epigenetic profiles, have distinct characteristics, compared to their technological predecessors, they do require a *new cycle of bio-identification process* before they can be granted any bio-objectification value. However, we believe that the original contribution that epigenetics offers to the bio-objectification framework is the possibility to include the environmental context in general, and the social context in particular, in what is *bio-objectifiable*. We thus propose the social context itself as target for bio-identification and as potential bio-object. Still our claim is not that account of the social put forward by epigenetics will and should simply replace all others accounts. Rather, it should be considered as complementary to non-molecular accounts as it offers new insights into relations between the social and the biological, visualizing how the social becomes molecularly embodied. That being said, the molecular embodiments of the social are: 1) sampled in the form of biological materials (entities); 2) recorded and measured in the form of epigenetic information (bio-virtual); 3) transformed into digital read-outs (bio-virtual with potential for bio-objectification), 3) archived in the form of both biological materials and digital read-outs, and 4) manipulated for research and/or technology-output purposes.

“The social” in epigenetics runs across all dimensions in bio-objectification and can indeed be considered as a bio-objectifiable itself. Moreover, some of the concerns were raised to the inclusion of “the social” in epigenetics. We would like to invite further investigation of epigenetics through bio-objectification framework in tracing and analyzing interrelatedness, transformation and movement of its research outputs in general, and of the social context in particular. We propose recent discussion within the bio-objects network and bio-objectification framework on European policy and economy with respect to the value of bio-objects and the new bio-economies they produce (18,19) as one direction in which this investigation of the social and epigenetics can be furthered.
